# Clinicopathological and preoperative biomarkers associated with recurrence after primary surgical treatment for endometrial cancer

**DOI:** 10.3389/fonc.2026.1882368

**Published:** 2026-09-03

**Authors:** Yi-Hua Liu, Bing Wei

**Affiliations:** 1 Department of Obstetrics and Gynecology, The Second Affiliated Hospital of Anhui Medical University, Hefei, Anhui, China; 2 Department of Obstetrics and Gynecology, Anqing 116th Hospital, Anqing, Anhui, China

**Keywords:** cancer antigen 125, endometrial cancer, fibrinogen, neutrophil-to-lymphocyte ratio, primary surgical treatment, recurrence

## Abstract

**Background:**

Postoperative recurrence remains a major concern in endometrial cancer (EC), and routinely available preoperative biomarkers may complement established clinicopathological factors. This study aimed to evaluate the associations of preoperative neutrophil-to-lymphocyte ratio (NLR), fibrinogen (FIB), and serum cancer antigen 125 (CA125) with recurrence after primary surgical treatment for EC.

**Methods:**

This single-center retrospective cohort included 368 patients treated between January 2019 and December 2024. Cohort-specific biomarker thresholds were derived by receiver operating characteristic analysis using the maximum Youden index. Multivariable logistic regression evaluated associations with recurrence after adjustment for International Federation of Gynecology and Obstetrics (FIGO) stage, histological type, and myometrial invasion. Sensitivity analyses, recurrence-free survival analysis, Firth penalized regression, and bootstrap internal validation were also performed.

**Results:**

During a median follow-up of 30 months, 32 patients (8.7%) experienced recurrence. Recurrence was associated with NLR >2.16 (odds ratio [OR], 4.03; 95% confidence interval [CI], 1.40–11.63), FIB >3.38 g/L (OR, 3.55; 95% CI, 1.30–9.67), CA125 >18.06 U/mL (OR, 2.52; 95% CI, 1.25–5.07), FIGO stage III disease (OR, 3.73; 95% CI, 1.45–9.59), non-adenocarcinoma histology (OR, 3.28; 95% CI, 1.35–7.96), and myometrial invasion ≥1/2 (OR, 3.01; 95% CI, 1.30–6.96). After Bonferroni correction, only FIGO stage III remained significant, whereas all six variables met the Benjamini–Hochberg false discovery rate threshold. The areas under the curve were 0.714 for NLR, 0.658 for FIB, and 0.765 for CA125. The optimism-corrected C-statistic was 0.835.

**Conclusion:**

Preoperative NLR, FIB, and CA125 were associated with postoperative recurrence and may provide complementary information to clinicopathological factors. The cohort-specific thresholds require external validation in larger multicenter studies before clinical application.

## 1 Introduction

Endometrial cancer (EC) is one of the most common gynecological malignancies worldwide, and its incidence has shown an increasing trend in recent years (1–3). Surgery remains the cornerstone of treatment for patients with clinically operable EC, and most patients with early-stage disease achieve favorable outcomes after radical surgical management (4, 5). However, a subset of patients still experience postoperative recurrence, including local pelvic recurrence and distant metastasis, which substantially compromises long-term survival and quality of life (6, 7). Therefore, accurate identification of patients at high risk of recurrence is essential for optimizing postoperative surveillance, adjuvant treatment decisions, and individualized disease management (8).

Postoperative recurrence in EC is a multifactorial process influenced by both tumor-related and host-related characteristics (9). Established clinicopathological factors, such as advanced Federation of Gynecology and Obstetrics (FIGO) stage, aggressive histological subtype, high tumor grade, and deep myometrial invasion, have been associated with unfavorable prognosis (10, 11). In addition, increasing attention has been paid to systemic inflammatory, coagulation-related, and tumor marker profiles, including the neutrophil-to-lymphocyte ratio (NLR), fibrinogen (FIB) level, and serum cancer antigen 125 (CA125) (12). These biomarkers are readily available in routine clinical practice and may provide prognostic information complementary to established clinicopathological factors (13, 14). However, whether preoperative laboratory markers improve recurrence risk assessment when considered alongside conventional clinicopathological characteristics remains insufficiently understood (15, 16). This uncertainty may hinder the identification of patients who could benefit from closer postoperative surveillance and more individualized follow-up strategies after primary surgical treatment (17).

To address this knowledge gap, we retrospectively analyzed patients with endometrial cancer who underwent primary surgical treatment at our institution. This study aimed to evaluate the associations of selected preoperative laboratory markers and clinicopathological characteristics with postoperative recurrence and to explore their potential value for recurrence risk stratification and follow-up planning.

## 2 Methods

### 2.1 Study design

This retrospective observational study included 368 patients who underwent primary surgical treatment for EC between January 2019 and December 2024. Primary surgical treatment consisted of total hysterectomy with or without bilateral salpingo-oophorectomy and, when clinically indicated, lymph node assessment or dissection. Pathological stage was assigned according to the 2014 International Federation of Gynecology and Obstetrics staging system, under which molecular classification was not incorporated into stage assignment. Patients were eligible if they had histologically confirmed EC, underwent primary surgical treatment as defined above, had sufficient clinical, pathological, and follow-up data for the primary analysis, provided informed consent for the use of their medical records for research purposes, and had a minimum postoperative follow-up period of 12 months for recurrence assessment. Patients were excluded if they had a concurrent or previous malignancy, received neoadjuvant chemotherapy or radiotherapy, had incomplete surgical resection, positive surgical margins, or residual disease at surgery, or had severe comorbid conditions that could substantially affect survival or follow-up, such as advanced cardiovascular disease or terminal illness. The study was designed and reported in accordance with the STROBE (Strengthening the Reporting of Observational Studies in Epidemiology) guidelines to ensure methodological rigor and transparency (18). The study protocol was reviewed and approved by the ethics committee of our hospital, and all procedures were conducted in accordance with the Declaration of Helsinki. Informed consent was obtained from all patients. All personal identifiers were removed before analysis to ensure data confidentiality and protect patient privacy.

### 2.2 Surgical staging and postoperative adjuvant treatment

Primary surgical treatment consisted of total hysterectomy with or without bilateral salpingo-oophorectomy. In routine clinical practice, the surgical approach and extent of staging were based on patient characteristics, preoperative evaluation, intraoperative findings, and the treating surgeon’s judgment. When performed, nodal assessment included sentinel lymph node mapping, pelvic lymph node dissection, or combined pelvic and para-aortic lymph node dissection, as clinically indicated. Peritoneal washing and omental assessment were performed selectively according to histological characteristics, suspected extrauterine disease, and intraoperative findings. Postoperative adjuvant treatment was selected after pathological review and clinical assessment, taking into account FIGO stage, histological type and grade, depth of myometrial invasion, nodal status, LVSI when available, and other relevant clinical factors. Radiotherapy consisted of vaginal brachytherapy, external-beam radiotherapy, or both. Details regarding chemotherapy regimens, number of cycles, combined-modality treatment, and treatment completion were retrospectively extracted from oncology treatment records.

### 2.3 Follow-up protocol

Patients were followed through outpatient visits and telephone consultations. The routine institutional follow-up schedule generally consisted of visits every three months during the first two postoperative years, every six months from the third to fifth postoperative years, and annually thereafter. The final follow-up date was June 25, 2026. Follow-up evaluations included medical history review, physical examination, laboratory testing, tumor marker assessment, and routine imaging. Additional computed tomography or magnetic resonance imaging was performed when clinically indicated to evaluate suspected recurrence or metastasis.

### 2.4 Criteria for recurrence evaluation

Recurrence was defined as the reappearance of malignancy related to the original EC after primary surgical treatment, including both local and distant recurrence. Local recurrence referred to tumor reemergence within the pelvic region, while distant metastasis indicated spread beyond the pelvis to organs such as the lungs, liver, or bones. The time to recurrence was calculated as the interval from the date of primary surgical treatment to the date of first documented recurrence, confirmed by histopathology or imaging. Patients without documented recurrence were censored at their last follow-up contact. All suspected recurrences were confirmed through either histopathological examination or advanced imaging modalities, including CT, MRI, or positron emission tomography (PET), when necessary.

### 2.5 Data collection

Data were retrospectively extracted from medical records, follow-up documentation, operative reports, pathological reports, molecular testing reports, and oncology treatment records. Recurrence-related information included recurrence status, recurrence site, and time to recurrence. Demographic and clinicopathological variables included age, body mass index, FIGO stage, histological type and grade, depth of myometrial invasion, hypertension, diabetes, and postoperative adjuvant radiotherapy and chemotherapy. Preoperative NLR, FIB, and CA125 values were obtained from the last available blood sample collected within 7 days before primary surgical treatment. When multiple measurements were available within this period, the measurement closest to the date of surgery was used. The NLR was calculated by dividing the absolute neutrophil count by the absolute lymphocyte count. FIB and CA125 levels were recorded in g/L and U/mL, respectively. The primary multivariable logistic regression model evaluated the associations of preoperative NLR, FIB, and serum CA125, together with FIGO stage, histological type, and depth of myometrial invasion, with postoperative recurrence. Because postoperative adjuvant treatment was determined after surgery and could be influenced by pathological risk factors, radiotherapy and chemotherapy were not included in the primary model but were additionally incorporated into a sensitivity analysis to assess the robustness of the primary findings. Additional surgical and treatment characteristics were collected for descriptive analyses. Surgical variables included surgical approach, bilateral salpingo-oophorectomy, type of nodal assessment, number of lymph nodes examined, pathological nodal status, peritoneal washing, and omental assessment. Postoperative treatment variables included the overall adjuvant treatment pattern, radiotherapy modality, chemotherapy regimen, number of chemotherapy cycles, and treatment completion status. LVSI was recorded as absent or present according to the original pathological reports. POLE mutation, mismatch repair, and p53 status were recorded as reported in the available pathological or molecular testing records. No retrospective molecular retesting was performed. Complete molecular classification was recorded only for patients with sufficient information for classification. Unperformed tests and unavailable results were coded as unavailable and were not interpreted as negative findings. Data availability for LVSI, POLE mutation status, MMR status, p53 status, and complete molecular classification was recorded separately for the overall cohort and according to recurrence status.

### 2.6 Statistical analysis

Statistical analyses were performed using IBM SPSS Statistics version 27.0 and R software. Continuous variables were summarized as mean ± standard deviation or median with interquartile range, as appropriate, and categorical variables as frequencies and percentages. Comparisons between the recurrence and non-recurrence groups were performed using Student’s t-test or the Mann–Whitney U test for continuous variables and the chi-square test or Fisher’s exact test for categorical variables, as appropriate. Receiver operating characteristic curve analysis was used to assess the ability of preoperative NLR, FIB, and CA125 to discriminate recurrence status. Exploratory cohort-specific cutoff values were determined using the maximum Youden index. Multivariable logistic regression was used to evaluate the associations of these biomarkers, FIGO stage, histological type, and depth of myometrial invasion with recurrence. Odds ratios and 95% confidence intervals were reported. Multiple testing across the six variables in the primary model was addressed using Bonferroni correction and the Benjamini–Hochberg false discovery rate procedure. Two sensitivity analyses were performed. First, the three biomarkers were entered into the multivariable logistic model as continuous variables. Second, postoperative radiotherapy and chemotherapy were added to the primary model. Model fit and calibration were assessed using the Hosmer–Lemeshow goodness-of-fit test, Brier score, and calibration plot. Recurrence-free survival was evaluated using Kaplan–Meier estimates and log-rank tests. An exploratory multivariable Cox regression analysis was performed using the same six variables as the primary logistic model, with hazard ratios and 95% confidence intervals reported. Model stability was assessed using the events-per-variable ratio, Firth penalized logistic regression, and bootstrap internal validation with 1,000 resamples. The bootstrap analysis estimated optimism-corrected discrimination, calibration slope, calibration intercept, and Brier score. *Post-hoc* power estimates were reported descriptively. The availability of LVSI and molecular data was summarized for the overall cohort and by recurrence status. Exploratory comparisons were restricted to patients with available data for each variable, using variable-specific denominators without imputation and the chi-square test or Fisher’s exact test, as appropriate. LVSI and molecular variables were not included in the primary multivariable model. Surgical staging and postoperative adjuvant treatment characteristics were summarized descriptively without inferential comparisons. All tests were two-sided, with P < 0.05 considered statistically significant unless otherwise specified.

## 3 Results

### 3.1 Comparison of clinical and pathological characteristics between recurrence and non-recurrence groups

A total of 368 patients with endometrial cancer were included, comprising 336 patients in the non-recurrence group and 32 patients in the recurrence group. Continuous age, BMI, age category, histological grade, hypertension, and diabetes did not differ significantly between the two groups (all P > 0.05). The recurrence group showed higher proportions of patients with NLR >2.16, FIB >3.38 g/L, serum CA125 >18.06 U/mL, FIGO stage III disease, non-adenocarcinoma histology, myometrial invasion ≥1/2, postoperative adjuvant radiotherapy, and postoperative adjuvant chemotherapy than the non-recurrence group (all P < 0.05). Detailed comparisons are presented in **Table 1**.

**Table 1:** Comparison of demographic, clinical, pathological, and treatment-related factors between non-recurrence and recurrence groups in patients with endometrial cancer.

Variable	Subcategory	Non-recurrence (n = 336), mean ± SD or n (%)	Recurrence (n = 32), mean ± SD or n (%)	Statistic	P value
Age, years	—	53.0 ± 15.7	54.2 ± 14.7	−0.439	0.663
BMI, kg/m^2^	—	24.7 ± 3.9	24.6 ± 4.0	0.135	0.893
Age	≤50 years	129 (38.4)	13 (40.6)	0.061	0.804
	>50 years	207 (61.6)	19 (59.4)		
Histological grade	Grade 1–2	298 (88.7)	26 (81.3)	1.537	0.250
	Grade 3	38 (11.3)	6 (18.8)		
Hypertension	Yes	62 (18.5)	7 (21.9)	0.225	0.636
	No	274 (81.5)	25 (78.1)		
Diabetes	Yes	23 (6.8)	3 (9.4)	0.285	0.594
	No	313 (93.2)	29 (90.6)		
NLR	≤2.16	206 (61.3)	13 (40.6)	5.188	0.022
	>2.16	130 (38.7)	19 (59.4)		
FIB, g/L	≤3.38	289 (86.0)	17 (53.1)	22.56	<0.001
	>3.38	47 (14.0)	15 (46.9)		
Serum CA125, U/mL	≤18.06	180 (53.6)	9 (28.1)	7.573	0.006
	>18.06	156 (46.4)	23 (71.9)		
FIGO stage	I–II	308 (91.7)	22 (68.8)	16.57	<0.001
	III	28 (8.3)	10 (31.3)		
Histological type	Adenocarcinoma	320 (95.2)	23 (71.9)	25.19	<0.001
	Non-adenocarcinoma	16 (4.8)	9 (28.1)		
Myometrial invasion	<1/2	307 (91.4)	18 (56.3)	34.92	<0.001
	≥1/2	29 (8.6)	14 (43.8)		
Postoperative adjuvant radiotherapy	Yes	34 (10.1)	19 (59.4)	57.5	<0.001
	No	302 (89.9)	13 (40.6)		
Postoperative adjuvant chemotherapy	Yes	128 (38.1)	23 (71.9)	13.78	0.0002
	No	208 (61.9)	9 (28.1)		

BMI, body mass index; FIGO, International Federation of Gynecology and Obstetrics; NLR, neutrophil-to-lymphocyte ratio; FIB, fibrinogen; CA125, cancer antigen 125; SD, standard deviation; χ^2^, chi-square statistic.

### 3.2 Determination of optimal preoperative cutoff values for CA125, FIB, and NLR

**Figure 1:**
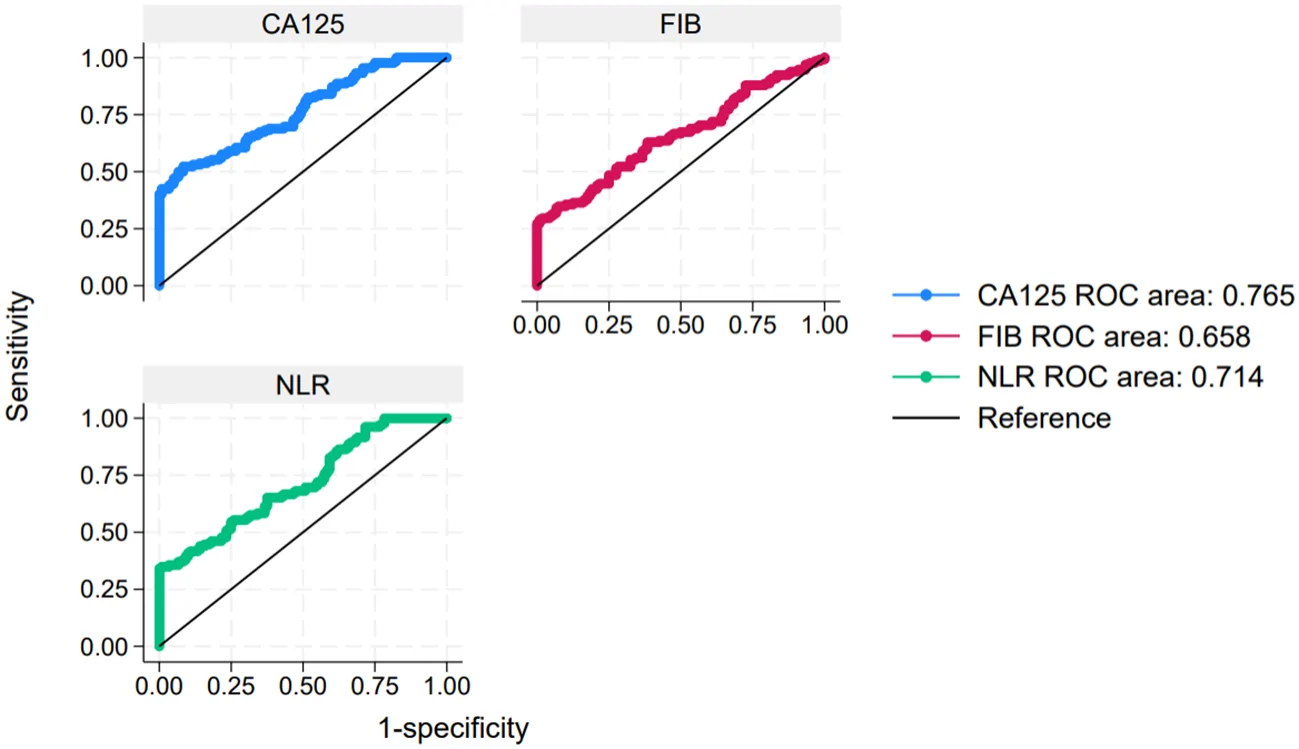
Receiver operating characteristic (ROC) curves of preoperative CA125, FIB, and NLR for discriminating postoperative recurrence in patients with endometrial cancer. The AUCs were 0.765 for CA125, 0.658 for FIB, and 0.714 for NLR. Detailed cutoff values, sensitivity, and specificity are provided in **Supplementary Table 1**.

ROC curve analysis was performed to determine the optimal cutoff values of preoperative serum CA125, FIB, and NLR for discriminating recurrence status in patients with EC. The maximum Youden index was used to identify the optimal thresholds. For CA125, the AUC was 0.765, and the optimal cutoff value was >18.06 U/mL, with a sensitivity of 71.9% and a specificity of 53.6%. For FIB, the AUC was 0.658, and the optimal cutoff value was >3.38 g/L, with a sensitivity of 46.9% and a specificity of 86.0%. For NLR, the AUC was 0.714, and the optimal cutoff value was >2.16, with a sensitivity of 59.4% and a specificity of 61.3%. These cutoff values were used consistently for the subsequent categorical analyses and multivariable logistic regression analysis. The detailed ROC performance of the three biomarkers is summarized in **Supplementary Table 1** and illustrated in **Figure 1**.

### 3.3 Multivariable logistic regression analysis of factors associated with recurrence in patients with endometrial cancer

Multivariable logistic regression analysis was performed to evaluate factors associated with recurrence in patients with EC. In the adjusted model, recurrence was associated with NLR >2.16 (OR = 4.033, 95% CI: 1.40–11.63, P = 0.0098), FIB >3.38 g/L (OR = 3.550, 95% CI: 1.30–9.67, P = 0.0134), and serum CA125 >18.06 U/mL (OR = 2.517, 95% CI: 1.25–5.07, P = 0.0097). Among clinicopathological variables, recurrence was associated with FIGO stage III disease (OR = 3.729, 95% CI: 1.45–9.59, P = 0.0063), non-adenocarcinoma histology (OR = 3.277, 95% CI: 1.35–7.96, P = 0.0088), and myometrial invasion ≥1/2 (OR = 3.008, 95% CI: 1.30–6.96, P = 0.0101) (**Table 2**).

**Table 2:** Multivariable logistic regression analysis of factors associated with recurrence in patients with endometrial cancer.

Factors	β coefficient	Standard error	Wald χ^2^	OR	95% CI	P value
NLR >2.16	1.395	0.540	6.67	4.033	1.40–11.63	0.0098
FIB >3.38 g/L	1.266	0.512	6.12	3.550	1.30–9.67	0.0134
Serum CA125 >18.06 U/mL	0.923	0.357	6.69	2.517	1.25–5.07	0.0097
FIGO Stage III	1.316	0.482	7.45	3.729	1.45–9.59	0.0063
Non-adenocarcinoma	1.187	0.453	6.87	3.277	1.35–7.96	0.0088
Myometrial invasion ≥1/2	1.101	0.428	6.62	3.008	1.30–6.96	0.0101

NLR, neutrophil-to-lymphocyte ratio; FIB, fibrinogen; CA125, cancer antigen 125; FIGO, International Federation of Gynecology and Obstetrics; OR, odds ratio; CI, confidence interval.

After adjustment for multiple testing across the six variables in the multivariable model, FIGO stage III remained statistically significant according to the Bonferroni-adjusted threshold (α = 0.00833). All six variables had Benjamini-Hochberg false discovery rate–adjusted q-values <0.05, as shown in **Supplementary Table 2**.

### 3.4 Model fit and calibration

Model fit and calibration were evaluated for the primary multivariable logistic regression model. The Hosmer–Lemeshow goodness-of-fit test showed no evidence of poor model fit (χ^2^ = 6.33, df = 8, P = 0.610). The Brier score was 0.070, indicating low overall prediction error for recurrence probability. The calibration plot showed that the predicted recurrence probabilities were generally consistent with the observed recurrence rates across deciles of predicted risk, with mild deviation in the intermediate-risk range. These findings indicated no obvious evidence of poor calibration in the primary logistic regression model. Detailed observed and expected recurrence rates across predicted-risk deciles are presented in **Supplementary Table 3**, and the calibration plot is shown in **Supplementary Figure 1**.

### 3.5 Sensitivity analysis

Additional sensitivity analyses were performed to evaluate the robustness of the primary multivariable logistic regression model. First, NLR, FIB, and serum CA125 were entered into the multivariable logistic regression model as continuous variables, while the same clinicopathological covariates as those in the primary model were retained. In this model, NLR per 1-unit increase (OR = 1.619, 95% CI: 1.17–2.24, P = 0.0035), FIB per 1-g/L increase (OR = 1.737, 95% CI: 1.13–2.67, P = 0.0117), and serum CA125 per 10-U/mL increase (OR = 1.114, 95% CI: 1.03–1.21, P = 0.0084) were statistically associated with recurrence. FIGO stage III (OR = 3.473, 95% CI: 1.33–9.06, P = 0.0109), non-adenocarcinoma histology (OR = 3.019, 95% CI: 1.22–7.45, P = 0.0165), and myometrial invasion ≥1/2 (OR = 2.773, 95% CI: 1.18–6.49, P = 0.0188) were also statistically associated with recurrence. These results are shown in **Supplementary Table 4**.

Second, postoperative adjuvant radiotherapy and chemotherapy were additionally included in the multivariable logistic regression model. In this model, the variables included in the primary model remained statistically associated with recurrence: NLR >2.16 (OR = 3.914, 95% CI: 1.33–11.54, P = 0.0133), FIB >3.38 g/L (OR = 3.302, 95% CI: 1.17–9.34, P = 0.0242), serum CA125 >18.06 U/mL (OR = 2.386, 95% CI: 1.14–4.99, P = 0.0209), FIGO stage III (OR = 3.120, 95% CI: 1.05–9.25, P = 0.0404), non-adenocarcinoma histology (OR = 2.941, 95% CI: 1.12–7.73, P = 0.0286), and myometrial invasion ≥1/2 (OR = 2.742, 95% CI: 1.10–6.83, P = 0.0304). Postoperative adjuvant radiotherapy (OR = 1.682, 95% CI: 0.57–4.97, P = 0.3466) and chemotherapy (OR = 1.244, 95% CI: 0.46–3.36, P = 0.6669) were not statistically significant in this sensitivity model. These results are shown in **Supplementary Table 5**.

### 3.6 Recurrence-free survival analysis

An additional exploratory recurrence-free survival (RFS) analysis was performed using recurrence-time and follow-up-time information. The median follow-up duration was 30.0 months. During follow-up, 32 patients experienced recurrence, whereas 336 patients were censored at their last follow-up visit.

Kaplan–Meier analysis showed that patients with NLR >2.16 had shorter RFS than those with NLR ≤2.16 (log-rank P = 0.006). Shorter RFS was also observed in patients with FIB >3.38 g/L compared with those with FIB ≤3.38 g/L (log-rank P = 0.010), and in patients with serum CA125 >18.06 U/mL compared with those with CA125 ≤18.06 U/mL (log-rank P = 0.012) (**Supplementary Figure 2**).

Because only 32 recurrence events were available, the multivariable Cox proportional hazards model was considered exploratory and was performed as a sensitivity analysis using the same six variables as the primary logistic regression model. In this model, NLR >2.16 (HR = 2.768, 95% CI: 1.30–5.88, P = 0.0082), FIB >3.38 g/L (HR = 2.562, 95% CI: 1.18–5.56, P = 0.0171), serum CA125 >18.06 U/mL (HR = 2.195, 95% CI: 1.08–4.45, P = 0.0295), FIGO stage III (HR = 3.020, 95% CI: 1.42–6.43, P = 0.0042), non-adenocarcinoma histology (HR = 2.670, 95% CI: 1.23–5.81, P = 0.0133), and myometrial invasion ≥1/2 (HR = 2.455, 95% CI: 1.18–5.11, P = 0.0164) were associated with shorter RFS. The detailed Cox regression results are shown in **Supplementary Table 6**.

### 3.7 Model stability and descriptive *post-hoc* power analysis

Model stability was further assessed because the primary multivariable logistic regression model included six predictors and 32 recurrence events, corresponding to an events-per-variable ratio of 5.3. This value was below the traditional 10-events-per-variable rule, indicating that the regression estimates should be interpreted cautiously. Therefore, additional stability analyses were performed. Firth penalized logistic regression was conducted using the same six predictors as the primary model. The direction and magnitude of the penalized estimates were generally consistent with those of the primary logistic regression model. NLR >2.16, FIB >3.38 g/L, serum CA125 >18.06 U/mL, FIGO stage III, non-adenocarcinoma histology, and myometrial invasion ≥1/2 remained statistically associated with recurrence in the penalized model. Bootstrap internal validation with 1,000 resamples showed an apparent C-statistic of 0.866 and an optimism-corrected C-statistic of 0.835. The optimism-corrected calibration slope was 0.860, suggesting mild-to-moderate overfitting. The optimism-corrected Brier score was 0.076, indicating low overall prediction error after correction. These results are summarized in **Supplementary Table 7**. *Post-hoc* power analysis was retained as descriptive supplementary information for the six variables associated with recurrence in the primary multivariable logistic regression model, including NLR, FIB, CA125, FIGO stage, histological type, and myometrial invasion depth. Based on the observed sample size, recurrence distribution, observed effect sizes, and a two-sided significance level of α = 0.05, the average estimated *post-hoc* power across these six variables was 87.4%.

### 3.8 Exploratory analysis of LVSI and molecular characteristics

LVSI documentation was available for 310 of 368 patients (84.2%). POLE mutation status, MMR status, p53 status, and complete molecular classification were available for 152 (41.3%), 224 (60.9%), 220 (59.8%), and 144 (39.1%) patients, respectively. The proportions of available data were broadly similar between the recurrence and non-recurrence groups (**Supplementary Table 8**). Among patients with available data, LVSI was more frequent in the recurrence group than in the non-recurrence group (48.1% vs. 18.7%, P = 0.001). Abnormal p53 status was also more frequent in the recurrence group (31.6% vs. 10.4%, P = 0.017). No statistically significant between-group differences were observed for POLE mutation status, MMR status, or the overall distribution of molecular classification. Given the incomplete availability of molecular data and the limited number of recurrence events within these subsets, these analyses were considered exploratory (**Supplementary Table 9**).

### 3.9 Surgical staging and postoperative adjuvant treatment characteristics

Minimally invasive surgery was performed in 74.5% of patients, bilateral salpingo-oophorectomy in 95.7%, and nodal assessment in 302 patients. Pelvic lymph node dissection alone was the most common nodal staging procedure. The recurrence group showed higher proportions of open surgery, combined pelvic and para-aortic lymph node dissection, pathological nodal involvement, and omental assessment. Overall, 56.0% of patients received no postoperative adjuvant treatment, 3.0% received radiotherapy alone, 29.6% received chemotherapy alone, and 11.4% received both modalities. Carboplatin plus paclitaxel was the predominant chemotherapy regimen, with a median of six cycles. These characteristics were summarized descriptively without inferential comparison (**Supplementary Table 10**).

## 4 Discussion

This study evaluated preoperative inflammatory, coagulation, and tumor markers together with clinicopathological characteristics in relation to postoperative recurrence in patients with endometrial cancer. NLR, FIB, and serum CA125 are routinely available before surgery and may provide additional information for preoperative risk assessment (19–21). In the primary multivariable analysis, these biomarkers, together with FIGO stage III disease, non-adenocarcinoma histology, and myometrial invasion ≥1/2, were associated with recurrence. The consistency and stability of these associations were further examined using continuous-variable modeling, additional adjustment for postoperative adjuvant treatment, recurrence-free survival analysis, multiple-testing correction, model calibration, Firth penalized regression, and bootstrap internal validation. FIGO stage III remained statistically significant under the stricter Bonferroni threshold, whereas all six variables remained below the Benjamini–Hochberg false discovery rate threshold. Given the limited number of recurrence events and the exploratory nature of several analyses, these findings should be interpreted as hypothesis-generating. After independent validation, the identified factors may contribute to recurrence risk stratification and individualized postoperative follow-up planning (22, 23).

FIB showed a different discrimination pattern, with a modest AUC of 0.658 and a specificity of 86.0%. Elevated FIB may therefore characterize a smaller subgroup with a higher observed recurrence frequency rather than function as a sensitive screening marker. This pattern is biologically plausible given the involvement of FIB in coagulation activation, tumor-associated inflammation, extracellular matrix remodeling, and interactions between tumor cells and the vascular microenvironment (24). NLR showed intermediate discrimination, with an AUC of 0.714 and relatively balanced sensitivity and specificity, consistent with its role as a nonspecific marker of systemic inflammation. Associations of NLR, FIB, and CA125 with recurrence were also observed when these biomarkers were modeled continuously, reducing concern that the findings were entirely attributable to data-driven dichotomization. Nevertheless, the optimal thresholds for FIB and NLR were also derived from the present cohort and may not be transportable across populations or laboratory settings without external validation. Collectively, these biomarkers may reflect different and potentially complementary aspects of recurrence-related disease characteristics, but their incremental clinical value beyond established pathological and molecular factors remains to be determined (25, 26).

The clinicopathological findings were generally consistent with recognized patterns of endometrial cancer progression. FIGO stage III showed the most statistically robust association with recurrence and was the only variable that remained significant under the Bonferroni-adjusted threshold. This finding is consistent with the established importance of extrauterine disease extension in recurrence assessment and indicates that biomarker findings should be interpreted within the broader context of disease stage. Non-adenocarcinoma histology was also associated with recurrence, consistent with the more unfavorable clinical behavior reported for several non-endometrioid and uncommon histological subtypes. Myometrial invasion ≥1/2 was another pathological feature associated with recurrence (27, 28). and may reflect greater local invasiveness and a higher likelihood of lymphatic or extrauterine spread. The recurrence-free survival analyses provided additional, although exploratory, support for the primary findings. Elevated NLR, FIB, and CA125 were associated with shorter recurrence-free survival in Kaplan–Meier analyses, and the exploratory Cox model produced directionally consistent associations for the six variables included in the primary logistic model (29, 30). These time-to-event findings provide complementary context but require cautious interpretation because of the limited number of recurrence events.

The handling of postoperative adjuvant treatment also requires careful interpretation. Postoperative radiotherapy and chemotherapy were not included in the primary multivariable model because the study focused on preoperative biomarkers and clinicopathological factors associated with recurrence and was not designed to estimate treatment effects. Adjuvant treatment is selected after surgical and pathological assessment and is commonly guided by adverse disease characteristics, including advanced FIGO stage, aggressive histology, deep myometrial invasion, nodal involvement, and LVSI when available. Consequently, the observed relationship between adjuvant treatment and recurrence may largely reflect confounding by indication and underlying disease severity. To examine whether adjustment for postoperative treatment altered the principal estimates, radiotherapy and chemotherapy were added to a sensitivity model. The direction and magnitude of the associations for the biomarkers and clinicopathological factors remained generally consistent. However, this analysis cannot eliminate residual confounding or account fully for differences in treatment modality, regimen, duration, and completion. Accordingly, the findings should not be interpreted as evidence regarding the effectiveness of postoperative adjuvant treatment.

The strengths of this study include detailed clinicopathological and follow-up data and the evaluation of routinely available preoperative biomarkers in relation to postoperative recurrence in endometrial cancer. The observed associations were assessed using complementary statistical approaches, including multivariable logistic regression, sensitivity analyses, recurrence-free survival analysis, model calibration, Firth penalized regression, and bootstrap internal validation. These analyses provided complementary evidence regarding the consistency, calibration, and stability of the findings. Accordingly, this study provides a basis for further evaluating whether inflammatory, coagulation, and tumor marker profiles add clinically relevant information to established clinicopathological factors for recurrence risk stratification and postoperative follow-up planning. Several limitations should also be considered. First, the retrospective single-center observational design is susceptible to selection bias, information bias, institutional practice patterns, and residual confounding; therefore, the observed associations should not be interpreted as causal. Second, only 32 patients experienced recurrence, resulting in a low events-per-variable ratio. Although Firth penalized regression and bootstrap internal validation were performed, the limited number of events may still have contributed to unstable estimates, relatively wide confidence intervals, and residual overfitting. Variation in follow-up duration and retrospective ascertainment of recurrence timing may also have reduced the precision of the time-to-event analyses. Third, LVSI and molecular data, including POLE, MMR, and p53 status, were incomplete and were therefore evaluated only in exploratory available-case analyses rather than incorporated into the primary multivariable model. This may have resulted in residual confounding and may limit interpretation within contemporary molecularly informed prognostic frameworks. In addition, staging was based on the 2014 FIGO system, and complete molecularly informed restaging under the 2023 framework was not feasible for the entire cohort. Finally, the NLR, FIB, and CA125 thresholds were derived and evaluated in the same cohort using the maximum Youden index and should therefore be regarded as exploratory, cohort-specific values rather than validated clinical cutoffs. Their performance may vary across patient populations, laboratory methods, treatment patterns, and follow-up practices. Future prospective multicenter studies with larger cohorts, standardized laboratory and pathological procedures, complete LVSI and molecular classification data, uniform follow-up, and independent external validation are needed to confirm these findings and determine whether these biomarkers provide incremental prognostic value beyond established clinicopathological and molecular factors.

## 5 Conclusions

In this retrospective study, higher preoperative NLR, FIB, and cancer antigen 125 levels, together with FIGO stage III disease, non-adenocarcinoma histology, and myometrial invasion ≥1/2, were associated with postoperative recurrence after primary surgical treatment for endometrial cancer. These routinely available biomarkers may provide complementary information to established clinicopathological factors for recurrence risk stratification and postoperative follow-up planning. However, these findings and the cohort-specific biomarker thresholds require validation in larger independent multicenter cohorts before clinical application.

## Data Availability

The raw data supporting the conclusions of this article will be made available by the authors, without undue reservation.
