# Supplementary material for: Clinicopathological and preoperative biomarkers associated with recurrence after primary surgical treatment for endometrial cancer
Source: Front Oncol. 2026 Sep 3;16:1882368. doi: 10.3389/fonc.2026.1882368 (PMC13581802; doi:10.3389/fonc.2026.1882368)
Supplement: Supplementary file 1 [file Table1.docx]

Supplementary Table S1. ROC performance of preoperative biomarkers for discriminating recurrence after primary surgical treatment for endometrial cancer.

| Biomarker | AUC | Cutoff | Sensitivity | Specificity |
| --- | --- | --- | --- | --- |
| CA125 | 0.765 | >18.06 U/mL | 71.90% | 53.60% |
| FIB | 0.658 | >3.38 g/L | 46.90% | 86.00% |
| NLR | 0.714 | >2.16 | 59.40% | 61.30% |

Abbreviations: ROC, receiver operating characteristic; AUC, area under the curve; CA125, cancer antigen 125; FIB, fibrinogen; NLR, neutrophil-to-lymphocyte ratio.

Supplementary Table S2. Multiple testing correction for six variables included in the multivariable logistic regression model.

| Variable | Original p-value | Bonferroni-adjusted p-value | Significant after Bonferroni (α=0.00833) | Benjamini-Hochberg q-value |
| --- | --- | --- | --- | --- |
| FIGO stage III | 0.0063 | 0.0378 | Yes | <0.05 |
| Non-adenocarcinoma histology | 0.0088 | 0.0528 | No | <0.05 |
| CA125 >18.06 U/mL | 0.0097 | 0.0582 | No | <0.05 |
| NLR >2.16 | 0.0098 | 0.0588 | No | <0.05 |
| Myometrial invasion ≥1/2 | 0.0101 | 0.0606 | No | <0.05 |
| FIB >3.38 g/L | 0.0134 | 0.0804 | No | <0.05 |

Note: Bonferroni-adjusted significance was defined as α = 0.00833.

Abbreviations: FIGO, International Federation of Gynecology and Obstetrics; CA125, cancer antigen 125; NLR, neutrophil-to-lymphocyte ratio; FIB, fibrinogen; BH, Benjamini-Hochberg; q-value, false discovery rate–adjusted p-value; α, significance threshold.

Supplementary Table S3. Calibration assessment of the primary multivariable logistic regression model.

A. Calibration by predicted-risk deciles

| Predicted-risk decile | No. of patients | Mean predicted probability | Expected recurrence events | Observed recurrence events | Observed recurrence rate |
| --- | --- | --- | --- | --- | --- |
| 1 | 37 | 0.004 | 0.15 | 0 | 0.000 |
| 2 | 37 | 0.009 | 0.33 | 1 | 0.027 |
| 3 | 37 | 0.015 | 0.56 | 0 | 0.000 |
| 4 | 37 | 0.025 | 0.93 | 2 | 0.054 |
| 5 | 37 | 0.038 | 1.41 | 0 | 0.000 |
| 6 | 37 | 0.055 | 2.04 | 3 | 0.081 |
| 7 | 37 | 0.08 | 2.96 | 2 | 0.054 |
| 8 | 37 | 0.12 | 4.44 | 6 | 0.162 |
| 9 | 36 | 0.18 | 6.48 | 6 | 0.167 |
| 10 | 36 | 0.349 | 12.56 | 12 | 0.333 |
| Overall | 368 | 0.087 | 31.85 | 32 | 0.087 |

B. Overall model-fit indices

| Index | Value | Interpretation |
| --- | --- | --- |
| Hosmer–Lemeshow χ² | 6.33 | No evidence of poor fit |
| Degrees of freedom | 8 | — |
| Hosmer–Lemeshow P value | 0.610 | No evidence of poor fit |
| Brier score | 0.07 | Acceptable overall prediction error |
| Mean predicted probability | 0.087 | Close to observed recurrence rate |
| Observed recurrence rate | 0.087 | 32/368 patients |

Abbreviations: No., number; χ², chi-square statistic.

Supplementary Table S4. Multivariable logistic regression analysis with NLR, FIB, and CA125 modeled as continuous variables.

| Variable | β coefficient | Standard error | Wald χ² | OR | 95% CI | P value |
| --- | --- | --- | --- | --- | --- | --- |
| NLR, per 1-unit increase | 0.482 | 0.165 | 8.53 | 1.619 | 1.17–2.24 | 0.0035 |
| FIB, per 1-g/L increase | 0.552 | 0.219 | 6.35 | 1.737 | 1.13–2.67 | 0.0117 |
| Serum CA125, per 10-U/mL increase | 0.108 | 0.041 | 6.94 | 1.114 | 1.03–1.21 | 0.0084 |
| FIGO stage III | 1.245 | 0.489 | 6.48 | 3.473 | 1.33–9.06 | 0.0109 |
| Non-adenocarcinoma histology | 1.105 | 0.461 | 5.75 | 3.019 | 1.22–7.45 | 0.0165 |
| Myometrial invasion ≥1/2 | 1.02 | 0.434 | 5.52 | 2.773 | 1.18–6.49 | 0.0188 |

Abbreviations: NLR, neutrophil-to-lymphocyte ratio; FIB, fibrinogen; CA125, cancer antigen 125; FIGO, International Federation of Gynecology and Obstetrics; β, regression coefficient; χ², chi-square statistic; OR, odds ratio; CI, confidence interval.

Supplementary Table S5. Sensitivity analysis of multivariable logistic regression additionally including radiotherapy and chemotherapy.

| Factors | β coefficient | Standard error | Wald χ² | OR | 95% CI | P value |
| --- | --- | --- | --- | --- | --- | --- |
| NLR >2.16 | 1.365 | 0.551 | 6.13 | 3.914 | 1.33–11.54 | 0.0133 |
| FIB >3.38 g/L | 1.195 | 0.530 | 5.08 | 3.302 | 1.17–9.34 | 0.0242 |
| Serum CA125 >18.06 U/mL | 0.870 | 0.377 | 5.33 | 2.386 | 1.14–4.99 | 0.0209 |
| FIGO Stage III | 1.138 | 0.555 | 4.20 | 3.120 | 1.05–9.25 | 0.0404 |
| Non-adenocarcinoma | 1.079 | 0.493 | 4.79 | 2.941 | 1.12–7.73 | 0.0286 |
| Myometrial invasion ≥1/2 | 1.009 | 0.466 | 4.69 | 2.742 | 1.10–6.83 | 0.0304 |
| Radiotherapy | 0.520 | 0.552 | 0.89 | 1.682 | 0.57–4.97 | 0.3466 |
| Chemotherapy | 0.218 | 0.507 | 0.19 | 1.244 | 0.46–3.36 | 0.6669 |

Abbreviations: NLR, neutrophil-to-lymphocyte ratio; FIB, fibrinogen; CA125, cancer antigen 125; FIGO, International Federation of Gynecology and Obstetrics; β, regression coefficient; χ², chi-square statistic; OR, odds ratio; CI, confidence interval.

Supplementary Table S6. Sensitivity analysis using Cox proportional hazards regression for recurrence-free survival.

| Variable | β coefficient | Standard error | Wald χ² | HR | 95% CI | P value |
| --- | --- | --- | --- | --- | --- | --- |
| NLR >2.16 | 1.018 | 0.385 | 6.99 | 2.768 | 1.30–5.88 | 0.0082 |
| FIB >3.38 g/L | 0.941 | 0.394 | 5.70 | 2.562 | 1.18–5.56 | 0.0171 |
| Serum CA125 >18.06 U/mL | 0.786 | 0.361 | 4.74 | 2.195 | 1.08–4.45 | 0.0295 |
| FIGO stage III | 1.105 | 0.386 | 8.20 | 3.020 | 1.42–6.43 | 0.0042 |
| Non-adenocarcinoma histology | 0.982 | 0.397 | 6.12 | 2.670 | 1.23–5.81 | 0.0133 |
| Myometrial invasion ≥1/2 | 0.898 | 0.374 | 5.76 | 2.455 | 1.18–5.11 | 0.0164 |

Abbreviations: NLR, neutrophil-to-lymphocyte ratio; FIB, fibrinogen; CA125, cancer antigen 125; FIGO, International Federation of Gynecology and Obstetrics; β, regression coefficient; χ², chi-square statistic; HR, hazard ratio; CI, confidence interval.

Supplementary Table S7. Model stability assessment using EPV, Firth penalized logistic regression, and bootstrap internal validation.

A. Events-per-variable assessment

| Item | Value |
| --- | --- |
| Total sample size | 368 |
| Recurrence events | 32 |
| Non-recurrence cases | 336 |
| Predictors in the primary model | 6 |
| Events per variable | 5.3 |
| Interpretation | Below the traditional 10 EPV rule; estimates should be interpreted cautiously |

B. Firth penalized logistic regression using the same six predictors as the primary model

| Variable | Penalized β coefficient | Penalized OR | 95% profile likelihood CI | P value |
| --- | --- | --- | --- | --- |
| NLR >2.16 | 1.239 | 3.452 | 1.28–9.31 | 0.0146 |
| FIB >3.38 g/L | 1.126 | 3.083 | 1.17–8.12 | 0.0228 |
| Serum CA125 >18.06 U/mL | 0.831 | 2.296 | 1.14–4.62 | 0.0194 |
| FIGO stage III | 1.201 | 3.323 | 1.29–8.57 | 0.0129 |
| Non-adenocarcinoma histology | 1.071 | 2.918 | 1.18–7.23 | 0.0206 |
| Myometrial invasion ≥1/2 | 0.990 | 2.691 | 1.16–6.25 | 0.0217 |

C. Bootstrap internal validation of the primary logistic regression model, 1,000 resamples

| Performance index | Apparent value | Mean optimism | Optimism-corrected value | Interpretation |
| --- | --- | --- | --- | --- |
| C-statistic | 0.866 | 0.031 | 0.835 | Discrimination remained acceptable after correction |
| Calibration slope | 1.000 | 0.140 | 0.860 | Mild-to-moderate overfitting |
| Brier score | 0.070 | −0.006 | 0.076 | Low prediction error after correction |
| Calibration intercept | 0.000 | 0.018 | −0.018 | Minimal systematic miscalibration |

Abbreviations: EPV, events per variable; NLR, neutrophil-to-lymphocyte ratio; FIB, fibrinogen; CA125, cancer antigen 125; FIGO, International Federation of Gynecology and Obstetrics; β, regression coefficient; OR, odds ratio; CI, confidence interval; C-statistic, concordance statistic.

Supplementary Table S8. Availability of LVSI and molecular data in the study cohort

| Variable | Availability category | Overall cohort, n (%) | Non-recurrence group, n (%) | Recurrence group, n (%) |
| --- | --- | --- | --- | --- |
| LVSI documentation | Available | 310 (84.2) | 283 (84.2) | 27 (84.4) |
|  | Unavailable | 58 (15.8) | 53 (15.8) | 5 (15.6) |
| POLE mutation testing | Available | 152 (41.3) | 139 (41.4) | 13 (40.6) |
|  | Not tested or unavailable | 216 (58.7) | 197 (58.6) | 19 (59.4) |
| MMR assessment | Available | 224 (60.9) | 204 (60.7) | 20 (62.5) |
|  | Not tested or unavailable | 144 (39.1) | 132 (39.3) | 12 (37.5) |
| p53 assessment | Available | 220 (59.8) | 201 (59.8) | 19 (59.4) |
|  | Not tested or unavailable | 148 (40.2) | 135 (40.2) | 13 (40.6) |
| Complete molecular classification | Available | 144 (39.1) | 132 (39.3) | 12 (37.5) |
|  | Unavailable | 224 (60.9) | 204 (60.7) | 20 (62.5) |

Abbreviations: LVSI, lymphovascular space invasion; POLE, polymerase epsilon; MMR, mismatch repair.

Supplementary Table S9. Exploratory comparison of LVSI and molecular characteristics according to recurrence status among patients with available data

| Variable | Category | Overall among available cases, n/N (%) | Non-recurrence group, n/N (%) | Recurrence group, n/N (%) | P value |
| --- | --- | --- | --- | --- | --- |
| LVSI | Absent | 244/310 (78.7) | 230/283 (81.3) | 14/27 (51.9) | 0.001 |
|  | Present | 66/310 (21.3) | 53/283 (18.7) | 13/27 (48.1) |  |
| POLE status | No pathogenic POLE mutation | 137/152 (90.1) | 124/139 (89.2) | 13/13 (100.0) | 0.365 |
|  | Pathogenic POLE mutation | 15/152 (9.9) | 15/139 (10.8) | 0/13 (0.0) |  |
| MMR status | pMMR | 158/224 (70.5) | 145/204 (71.1) | 13/20 (65.0) | 0.61 |
|  | dMMR | 66/224 (29.5) | 59/204 (28.9) | 7/20 (35.0) |  |
| p53 status | Wild-type | 193/220 (87.7) | 180/201 (89.6) | 13/19 (68.4) | 0.017 |
|  | Abnormal | 27/220 (12.3) | 21/201 (10.4) | 6/19 (31.6) |  |
| Molecular classification | POLE-mutated | 14/144 (9.7) | 14/132 (10.6) | 0/12 (0.0) | 0.116 |
|  | MMR-deficient | 40/144 (27.8) | 37/132 (28.0) | 3/12 (25.0) |  |
|  | p53-abnormal | 17/144 (11.8) | 13/132 (9.8) | 4/12 (33.3) |  |
|  | NSMP | 73/144 (50.7) | 68/132 (51.5) | 5/12 (41.7) |  |

Abbreviations: LVSI, lymphovascular space invasion; POLE, polymerase epsilon; MMR, mismatch repair; pMMR, mismatch repair proficient; dMMR, mismatch repair deficient; NSMP, no specific molecular profile.

Supplementary Table S10. Surgical staging procedures and postoperative adjuvant treatment characteristics

| Domain | Variable | Category or summary measure | Overall cohort, n (%) or median (IQR) | Non-recurrence group, n (%) or median (IQR) | Recurrence group, n (%) or median (IQR) |
| --- | --- | --- | --- | --- | --- |
| Surgical procedure | Surgical approach | Minimally invasive | 274 (74.5) | 254 (75.6) | 20 (62.5) |
|  |  | Open | 89 (24.2) | 77 (22.9) | 12 (37.5) |
|  |  | Converted to open | 3 (0.8) | 3 (0.9) | 0 (0.0) |
|  |  | Unavailable | 2 (0.5) | 2 (0.6) | 0 (0.0) |
| Surgical procedure | Bilateral salpingo-oophorectomy | Yes | 352 (95.7) | 321 (95.5) | 31 (96.9) |
|  |  | No | 16 (4.3) | 15 (4.5) | 1 (3.1) |
| Nodal staging | Type of nodal assessment | No nodal assessment | 66 (17.9) | 64 (19.0) | 2 (6.3) |
|  |  | Sentinel lymph node mapping only | 82 (22.3) | 78 (23.2) | 4 (12.5) |
|  |  | Pelvic lymph node dissection only | 154 (41.8) | 143 (42.6) | 11 (34.4) |
|  |  | Pelvic and para-aortic lymph node dissection | 66 (17.9) | 51 (15.2) | 15 (46.9) |
| Nodal staging | Number of lymph nodes examined among patients undergoing nodal assessment | Median (IQR) | 14 (7–24), n=302 | 13 (6–22), n=272 | 22 (12–33), n=30 |
| Nodal staging | Pathological nodal status | Negative | 280 (76.1) | 258 (76.8) | 22 (68.8) |
|  |  | Positive | 22 (6.0) | 14 (4.2) | 8 (25.0) |
|  |  | Unknown or not assessed | 66 (17.9) | 64 (19.0) | 2 (6.3) |
| Additional staging | Peritoneal washing | Performed | 241 (65.5) | 220 (65.5) | 21 (65.6) |
|  |  | Not performed | 119 (32.3) | 109 (32.4) | 10 (31.3) |
|  |  | Unavailable | 8 (2.2) | 7 (2.1) | 1 (3.1) |
| Additional staging | Omental assessment | Performed | 63 (17.1) | 51 (15.2) | 12 (37.5) |
|  |  | Not performed | 294 (79.9) | 275 (81.8) | 19 (59.4) |
|  |  | Unavailable | 11 (3.0) | 10 (3.0) | 1 (3.1) |
| Adjuvant treatment | Overall treatment pattern | No adjuvant treatment | 206 (56.0) | 201 (59.8) | 5 (15.6) |
|  |  | Radiotherapy alone | 11 (3.0) | 7 (2.1) | 4 (12.5) |
|  |  | Chemotherapy alone | 109 (29.6) | 101 (30.1) | 8 (25.0) |
|  |  | Radiotherapy plus chemotherapy | 42 (11.4) | 27 (8.0) | 15 (46.9) |
| Radiotherapy | Radiotherapy modality among radiotherapy recipients | Vaginal brachytherapy | 22/53 (41.5) | 18/34 (52.9) | 4/19 (21.1) |
|  |  | External-beam radiotherapy | 20/53 (37.7) | 11/34 (32.4) | 9/19 (47.4) |
|  |  | Both modalities | 9/53 (17.0) | 4/34 (11.8) | 5/19 (26.3) |
|  |  | Modality unavailable | 2/53 (3.8) | 1/34 (2.9) | 1/19 (5.3) |
| Chemotherapy | Chemotherapy regimen among chemotherapy recipients | Carboplatin plus paclitaxel | 128/151 (84.8) | 108/128 (84.4) | 20/23 (87.0) |
|  |  | Other platinum-based regimen | 17/151 (11.3) | 14/128 (10.9) | 3/23 (13.0) |
|  |  | Other non-platinum regimen | 2/151 (1.3) | 2/128 (1.6) | 0/23 (0.0) |
|  |  | Regimen unavailable | 4/151 (2.6) | 4/128 (3.1) | 0/23 (0.0) |
| Chemotherapy | Number of chemotherapy cycles among chemotherapy recipients | Median (IQR) | 6 (4–6), n=151 | 6 (4–6), n=128 | 6 (3–6), n=23 |
| Treatment completion | Planned adjuvant treatment completed among patients with planned adjuvant treatment | Yes | 137/162 (84.6) | 113/135 (83.7) | 24/27 (88.9) |
|  |  | No | 20/162 (12.3) | 17/135 (12.6) | 3/27 (11.1) |
|  |  | Unavailable | 5/162 (3.1) | 5/135 (3.7) | 0/27 (0.0) |

Abbreviations: IQR, interquartile range.

Supplementary Figure S1. Calibration plot of the multivariable logistic regression model.

Supplementary Figure S2. Kaplan–Meier curves for recurrence-free survival according to preoperative biomarker status.

A: NLR ≤2.16 vs. >2.16

B: FIB ≤3.38 g/L vs. >3.38 g/L

C: CA125 ≤18.06 U/mL vs. >18.06 U/mL
